# Atomic-Scale Imaging of Polymers and Precision Molecular Weight Analysis

**DOI:** 10.1021/jacs.4c13812

**Published:** 2024-12-04

**Authors:** Arkadios Marathianos, Alexandros Magiakos, Yisong Han, Ana Sanchez, Richard Whitfield, Jochen Kammerer, Athina Anastasaki, Paul Wilson, Joseph P. Patterson, Christopher Barner-Kowollik, Evelina Liarou

**Affiliations:** † Polymer Characterization Research Technology Platform, University of Warwick, Coventry CV4 7AL, United Kingdom; ‡ Department of Chemistry, University of Warwick, Library Road, Coventry CV4 7AL, U.K.; § Department of Physics, University of Warwick, Coventry CV4 7AL, U.K.; ∥ Laboratory of Polymeric Materials, Department of Materials, ETH Zurich, Zurich 8093, Switzerland; ⊥ School of Chemistry and Physics, Centre for Materials Science, Queensland University of Technology (QUT), 2 George Street, Brisbane City, QLD 4000, Australia; # Department of Chemistry, University of California, Irvine, Irvine, California 92697-2025, United States; ◧ Department of Materials Science and Engineering, University of California, Irvine, Irvine, California 92697-2025, United States; ◪ Institute of Nanotechnology, Karlsruhe Institute of Technology (KIT), Kaiserstrasse 12, 76131 Karlsruhe, Germany

## Abstract

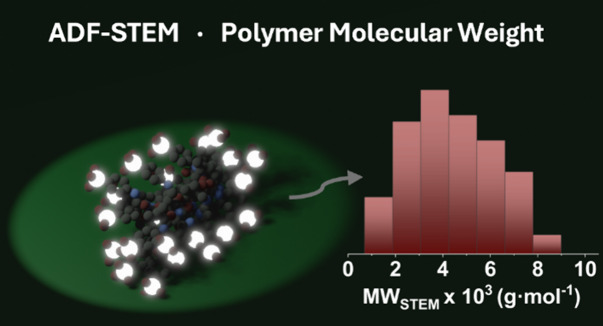

Polymer design requires fine control over syntheses and a thorough understanding of their macromolecular structure. Herein, near-atomic level imaging of polymers is achieved, enabling the precise determination of one of the most important macromolecular characteristics: molecular weight. By judiciously designing and synthesizing different linear metal(loid)-rich homopolymers, subnanoscale polymer imaging is achieved through annular dark field-scanning transmission electron microscopy (ADF-STEM), owing to the incorporation of high *Z* atoms in the side chain of the monomeric units. The molecular weight of these polymers can be precisely determined by detecting and counting their metal(loid) atoms upon ADF-STEM imaging, at sample concentrations as low as 10 μg·mL^–1^. Notably, a commonly used C, H, and O-containing polymer (*i.e.*, poly(methyl acrylate)) that was thus far inaccessible at the atomic scale is derivatized to allow for subnano-level imaging, thus expanding the scope of our approach toward the atomic-level visualization of commodity polymers.

The design of soft matter with predefined properties necessitates the (sub)nanoscale analysis of polymers, tailored with precision for significant performance.^1,2^ A fundamental characteristic of polymers is their molecular weight (MW). The leading techniques for MW determination are size exclusion chromatography (SEC), ^1^H nuclear magnetic resonance (^1^H NMR), and high-resolution mass spectrometry (HR-MS) (Scheme 1). Although well-established, these techniques possess significant limitations when complex systems are targeted, including organometallic^3,4^ and conjugated polymers,^5,6^ or complex architectures.^7,8^ For instance, SEC requires the combination of suitable solvents, columns and MW standards,^9^^1^H NMR requires distinctive end-groups, while topologically complex polymers, with high dispersity and MW, are not suitable for HR-MS.^10,11^

**Scheme 1:**
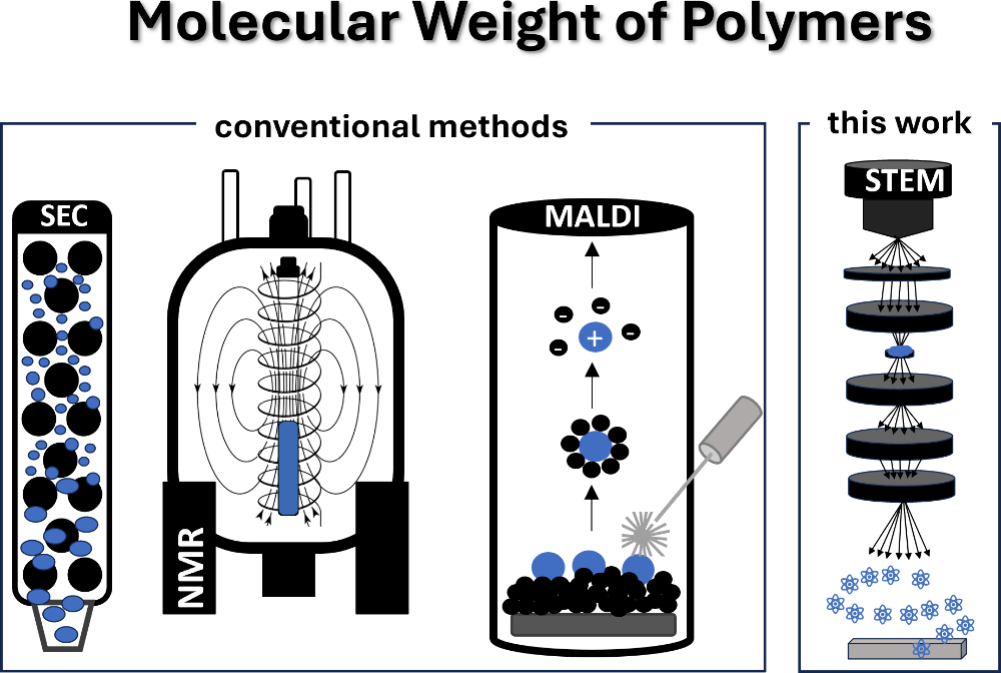
Schematic Illustration of the Methods Traditionally Used to Determine the MW of Polymers and Our Approach through ADF-STEM

Acknowledging those challenges, Junkers and colleagues developed a universal approach to determine polymer MW through diffusion-ordered NMR spectroscopy (DOSY), overcoming calibration and solvent implications,^12−14^ while Haddleton and Lester developed a facile strategy for MW online monitoring through DOSY.^15,16^ However, for polymers with compositional complexity and aggregation behavior in most solvents, solid-state MW analysis is necessary. Costantini and colleagues reported on the MW determination of conjugated polymers, through scanning tunnelling microscopy (STM) and vacuum electrospray deposition (ESD),^6^ while another powerful example is the work from Matyjaszewski and Sheiko who achieved in-depth analysis of high MW cylindrical brushes using atomic force microscopy (AFM).^17−19^ Although STM and AFM have provided valuable insights into the understanding of macromolecular characteristics, they are limited to the imaging of conjugated polymers or polymers with very high MW and branching.

Within the scope of visual understanding of polymers, electron microscopy (EM) techniques including (cryogenic) transmission electron microscopy, (Cryo-) TEM, and liquid-cell electron microscopy (LC-EM) have revolutionized the field of polymer imaging. Exemplary are the studies from Patterson,^1,20−26^ Gianneschi,^27−33^ Sommerdijk,^34,35^ and de Jonge,^36−40^ among others. However, the subnano level imaging of nonconjugated synthetic polymers has been largely inaccessible. Apart from their structural complexity, their elemental composition is mostly limited to C, O, H, and N, exhibiting similarity with most TEM support grids. Consequently, the low contrast obtained during conventional TEM does not allow for precise subnano level imaging. Additionally, their light element composition renders them challenging to detect through atomic-level EM methods, such as annular dark field (ADF) scanning transmission electron microscopy (STEM), where contrast depends on the atomic number.^1,41−44^

Our vision was to overcome those challenges and approach atomic-level analysis of polymers through ADF-STEM, as well as to visualize their MW (Scheme 1), by strategically designing the synthesis of metalloid-rich homopolymers bearing one arsenic (As) atom per monomer unit. For that purpose, free radical polymerization (FRP) and reversible deactivation radical polymerization (*i.e.*, reversible addition–fragmentation chain transfer polymerization, RAFT) were employed to generate polymers with various MW and *Đ* values. To expand to another polymer family and metal functionality, ferrocenylmethyl methacrylate was used to generate an Fe-rich polymethacrylate. Finally, to render widely used polymers visible on the atomic level, a poly(methyl acrylate) (PMA) was derivatized with ferrocenecarboxylic acid, and its MW and *Đ* were calculated through imaging.

Initially, an As-acrylamide monomer was synthesized according to the literature^45,46^ and used to generate an As-polyacrylamide (PAsAm) through FRP (PAsAm_FRP_, Figure S1, SI). A highly dilute (50 μg·mL^–1^ in 0.1 M NaOH) solution of the purified homopolymer was prepared and placed under vacuum prior to imaging (SI). To gain a first understanding of the As signal, we employed ADF-STEM through a double aberration corrected JEOL ARM200F microscope, operated at 200 kV. At 3 million times magnification (×3M), bright nanoclusters were evident (Figure S2), while at ×8M and ×12M magnification, their structure was elucidated, depicting the randomly coiled polymer chains consisting of As atoms (appearing as bright spots, Figure S2). To enhance sample stability and mitigate contamination, “beam shower” was applied prior to imaging at high magnifications.^47^ Although the organic content is sensitive and prone to beam damage under the applied conditions,^46^ the metalloid-rich chains remained intact throughout imaging. An advantage of this approach is that any potential damage to the organic components of the polymers by the electron beam will not affect the results of the MW analysis, as they depend only on the beam-stable metals.

**Figure 1:**
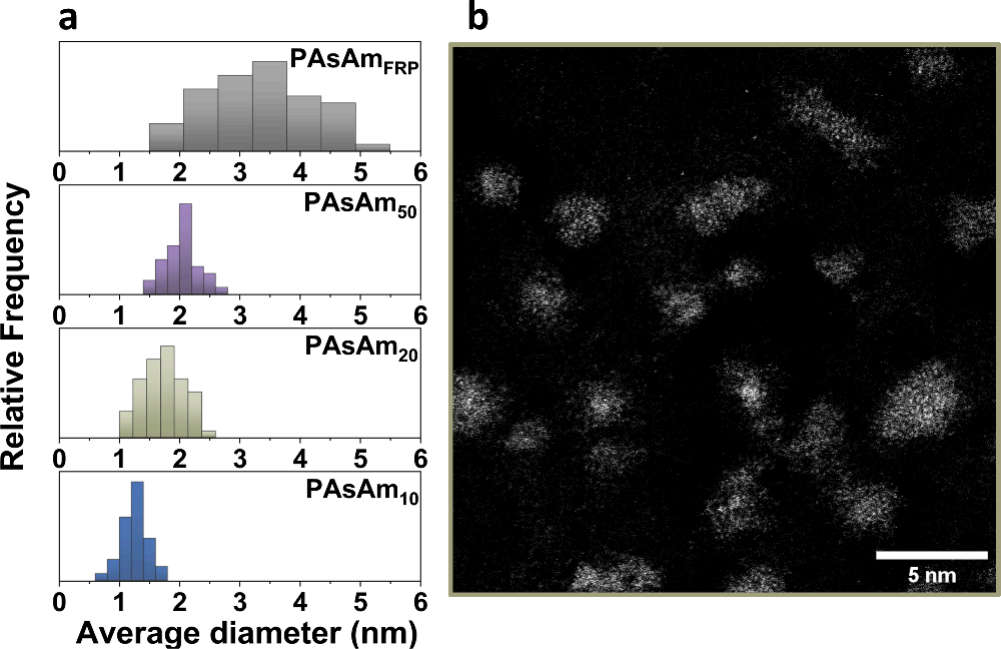
(a) Histograms showing the distribution of chain diameter for the four polymers and (b) high-resolution ADF-STEM image of PAsAm_20_ (scale bar: 5 nm).

Having achieved the detection of single chains and their As atoms, we sought to visualize the MW distribution of the polymers. Three well-defined PAsAms with targeted *DP*_n_ = 50, 20, and 10 were synthesized through RAFT polymerization (SI, Figures S3–S5), while aqueous-SEC and DOSY NMR were employed to determine the MW of the homopolymers after purification (Table 1).The ADF-STEM of PAsAm_50_, PAsAm_20_ (Figure 1b), and PAsAm_10_ at ×8M magnification revealed polymer chains smaller than in the case of PAsAm_FRP_ (Figures 1a, 2c–f, and S6–S8). Owing to the different average chain length of the imaged polymers, the chain diameter increased with the increase in MW (Figure 1a), while the low *Đ* polymers exhibited narrow diameter distribution, compared to PAsAm_FRP_. Importantly, when the As-monomer was imaged under the same EM conditions, only individually scattered single As atoms were detected (Figure S9).

**Table 1:** Molecular Weight and *Đ* Values from SEC, DOSY, and STEM for the Different Metal(loid)-rich Homopolymers

Polymer	*M*_n,SEC_^a^	*DP*_n,SEC_^a^	*M*_w,SEC_^a^	*Đ*_SEC_^a^	MW_DOSY_^b^	*DP*_n,DOSY_^b^	MW_STEM_^c^	*DP*_n,STEM_^c^	*Đ*_STEM_^c^,^d^
PAsAm_10_	5,700	21	6,300	1.10	2,500	9	2,300	8	1.30
PAsAm_20_	7,900	29	8,700	1.10	6,900	25	7,500	28	1.20
PAsAm_50_	12,100	45	13,900	1.15	9,600	35	11,000	40	1.10
PAsAm_FRP_	218,000	N/A	509,000	2.30	101,700	375	113,000^h^	417^h^	1.60
PFerMMA_10_	5,900^e^	21^e^	8,600^e^	1.4^e^,^f^	3,700^g^	14^g^	3,500	13	1.40
	7,100^f^	25^f^	10,200^f^						

a Aqueous-SEC, average molecular weight values expressed as MW equivalents relative to PEG/PEO standards,

b in D_2_O/NaOH using an 80 MHz benchtop NMR, calculated through MaDDOSY,^15^

c MW_STEM_ expressed in g·mol^–1^, conditions: 200 kV at ×8M magnification (and ×10M for PAsAm_10_ and PFerMMA_10_),

d calculated based on the literature,^51^

e CHCl_3_-SEC, average molecular weight values expressed as MW equivalents to PS or

f PMMA standards,

g in CDCl_3_ using an 80 MHz benchtop NMR,

h average of the broad main distribution from 13,000 to 80,000 g·mol^–1^ (*DP*_n_ = 48–295) and chains reaching up to 240,000 g·mol^–1^ (*DP*_n_ ∼ 885).

**Figure 2:**
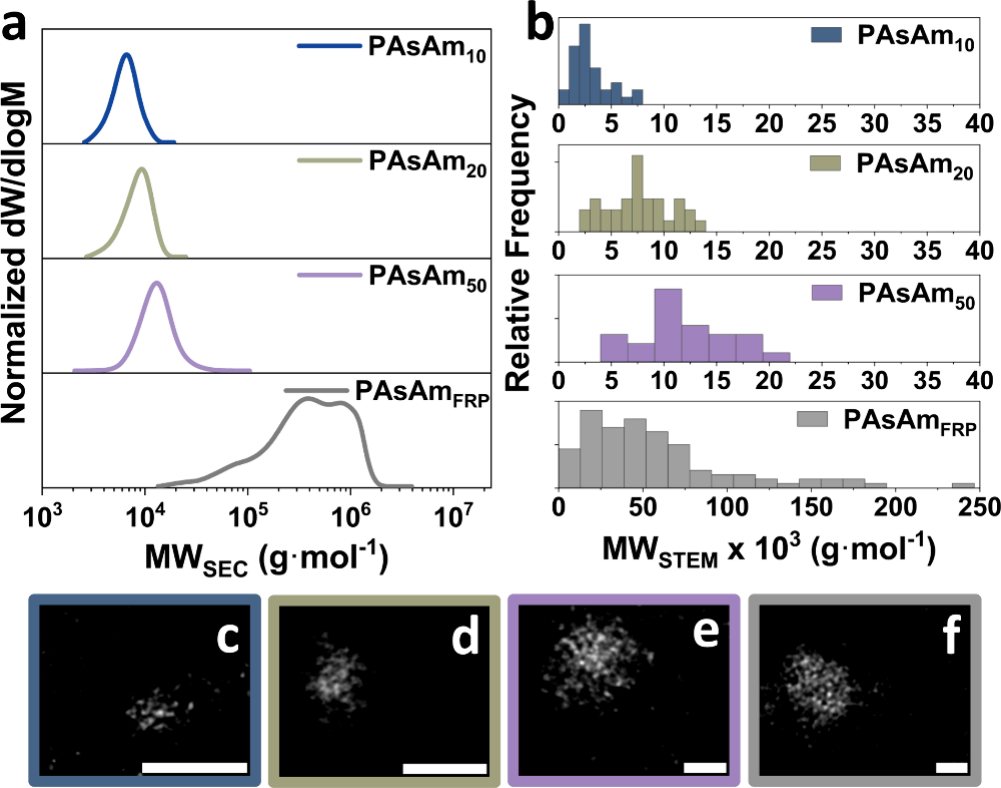
(a) Aqueous-SEC traces for PAsAm_10_, PAsAm_20_, PAsAm_50_ and PAsAm_FRP_. (b) MW_STEM_ distributions for the different polymers and ADF-STEM images showing segmented individual polymer chains for (c) PAsAm_10_, (d) PAsAm_20_, (e) PAsAm_50_, and (f) PAsAm_FRP_ (scale bars: 1 nm, *images were smoothed post imaging*).

To determine the polymer MW and *Đ*, the intensities of single chains were calculated upon subtraction of their background, while the intensity of single As atoms was used as the calibrant, assuming a linear relationship between the integrated ADF intensity of single atoms and very small nanoclusters when kinematic diffraction effects dominate the signals collected by ADF-STEM imaging (Figure S10).^48−50^ The same process was repeated for each sample individually in the same session. The integrated As atoms’ intensity allowed for determination of the *DP*_n_ for PAsAm_50_, PAsAm_20_, and PAsAm_10_ through atom counting (Table 1, SI).

In other words, the number of As atoms in each chain corresponded to *DP*_n_, which was used to calculate the corresponding MW_STEM_. The *Đ*_STEM_ of the polymers was estimated according to the literature, based on standard deviation (σ), and the relation between *Đ* and σ (SI).^51^

For the three well-defined polymers, MW_STEM_ was comparable with MW_SEC_, while there was particularly good agreement between STEM and DOSY for PAsAm_10_ and PAsAm_20_ (Table 1; Figures 2a–b, S4, S5, S7, and S8). Importantly, in contrast with DOSY, STEM can provide a distribution of MW, representative of the nonidentical chain lengths in a synthetic polymer sample. For PAsAm_10_, *M*_n,SEC_ was significantly higher than MW_DOSY_ because low MWs necessitate better separation for higher accuracy. The *Đ*_STEM_ results for PAsAm_20_ and PAsAm_50_ exhibited proximity to *Đ*_SEC_ with both approaches resulting in *Đ*_STEM_ ≤ 1.2.

To push the limits of our system, we attempted to calculate the MW_STEM_ of PAsAm_FRP_. As expected, the STEM results showed a nonsymmetrical distribution of As atoms per chain, with a predominant broad MW_STEM_ distribution from 13,000 to ∼80,000 g·mol^–1^, along with the presence of high MW species up to ∼240,000 g·mol^–1^, with average MW_STEM_ = 113,000 g·mol^–1^ (Figure 2b, Table 1). The *Đ*_STEM_ was 1.60, and although lower than the corresponding *Đ*_SEC_, it illustrated the broad MW distribution of PAsAm_FRP_. Samples with such high *DP*_n_ heterogeneity are highly challenging to quantitatively analyze with accuracy from single ADF-STEM images, since the very high MW chains might exhibit similarities with aggregated species; thus, careful interpretation of the images is necessary.^44,52^ In general, highly pure polymer samples, careful sample preparation (*i.e.*, suitable support grids),^53,54^ and thorough pretreatment (*i.e.*, vacuum drying, beam shower,^47,55^SI) are essential requirements, especially when sensitive samples are used.^53^

To expand the scope of metal functionality and polymer type, we synthesized an Fe-rich polymethacrylate (PFerMMA_10_) (Figures 3a and S11, SI). As in the case of PAsAms, the MW_STEM_ (Figure 3b,d,e) was comparable to MW_DOSY_ while lower than *M*_n,SEC_ (Table 1, Figure 3b,c). The SEC analysis of PFerMMA_10_ exhibited distinct deviations when PMMA and PS standards were used (Figure 3c), highlighting the limitations of SEC when samples deviate from the calibrant. The range of MW_STEM_ (∼1,000–12,000 g·mol^–1^) with the existence of a second smaller population with MW ∼9,500–12,000 g·mol^–1^, was depicted in the obtained *Đ*_STEM_ = 1.40 (Table 1). Therefore, the calculation of MW_STEM_ could be successfully achieved both for metalloid- and metal-containing acrylamide and methacrylate homopolymers, while their *DP*_n_ heterogeneity could be estimated through *Đ*_STEM_ calculation.

**Figure 3:**
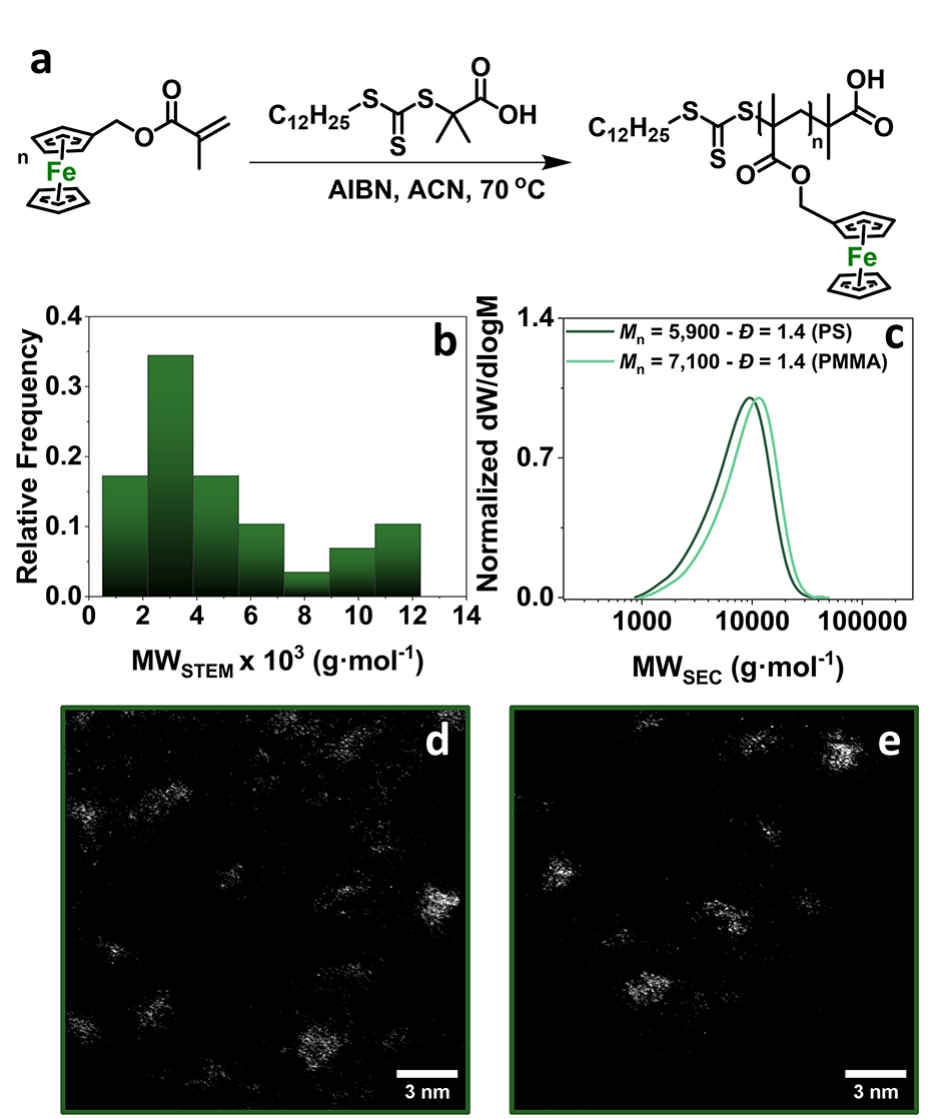
(a) Reaction scheme for the synthesis of PFerMMA_10_, (b) MW_STEM_ histogram for PFerMMA_10_, (c) SEC traces of PFerMMA_10_, and (d and e) ADF-STEM images for PFerMMA_10_ (scale bar: 3 nm).

**Figure 4:**
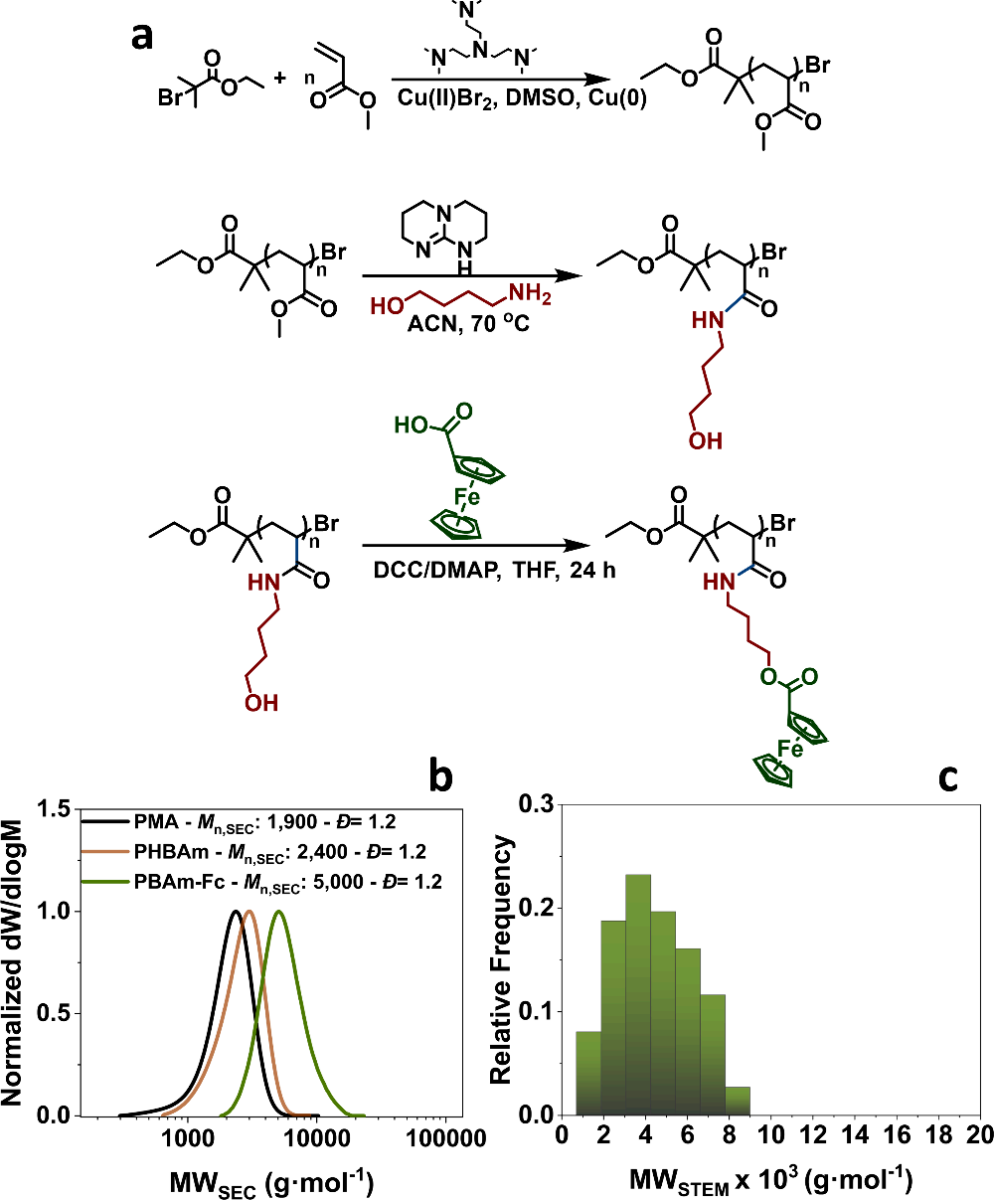
(a) Reaction scheme for the derivatization of PMA. (b) SEC traces of the parent PMA, the amidated derivative PHBAm, and the final PBAm-Fc, and (c) MW_STEM_ histogram of the functional PBAm-Fc.

Finally, we were interested in applying our approach to widely used C-, H-, and O-containing polymers, without using specially designed monomers. Thus, a PMA_20_ was synthesized (Figures 4a and S12, SI) and subsequently amidated using 4-amino-1-butanol, according to a literature procedure.^56^ The amidation of PMA_20_ to poly(hydroxybutyl acrylamide, PHBAm) was quantitative, with a full shift of the PMA methyl protons as verified by ^1^H NMR (Figure S14d), and full shift of the 1730 cm^–1^ peak (C=O, PMA) along with the formation of the 1635 cm^–1^ (C=O, amide) and 1543 cm^–1^ (N–H) PHBAm peaks, as verified by FT-IR (Figure S13). The shift toward higher MW was verified by THF-SEC (Figure 4b). The obtained −OH functional polymer was further functionalized through DCC/DMAP coupling with ferrocene (Fc) carboxylic acid (Figure 4a, SI), leading to derivatization of the parent PMA into an Fe-containing polyacrylamide. THF-SEC showed a clear shift toward higher MW (Figures 4b and S14); FT-IR verified the appearance of the C=O band (1700 cm^–1^) attributed to the Fc-ester (Figure S13), while ^1^H NMR confirmed the incorporation of the Fc moieties in the polymer (Figure S14d). ADF-STEM (Figure S15) revealed a predominant MW_STEM_ distribution at 3,000–4,300 g·mol^–1^ (*vs M*_n,SEC_ = 5,000, Figure 4b,c), indicating that on average ∼11 monomer units per chain had been functionalized (*vs* ∼14 from SEC). Therefore, the achievement of near-atomic level imaging of a commonly used polymer through derivatization has critical potential to serve as a promising strategy to visualize materials that had thus far been unobtainable. To the best of our knowledge, this is the first example of near-atomic level imaging of such a widely used polymer. Owing to the various synthetic tools available, we envisage that the modification of other commonly used polymers (*i.e.*, polystyrene, polyolefins) through different derivatization approaches (*i.e.*, Diels–Alder, click chemistry)^57−64^ will expand the scope of this approach and establish it as a platform for advanced polymer imaging.

Our work presents the first approach toward atomic level imaging of synthetic polymers and MW determination through atom counting. By combining metal(loid)-containing monomers, different polymerization approaches, and atom counting through ADF-STEM, fundamental polymer characteristics were determined in the subnano scale. Additionally, the subnano level imaging of a widely used polymer (*i.e.*, PMA) was achieved upon derivatization. Our combinatorial approach sets the ground for atomic level analysis of polymer fundamentals that could not be imaged with such precision before and facilitates the profound understanding of their structure–property relationships.

## Supporting Information Available

Materials, instrumentation details, methods, synthetic procedures, NMR, SEC, FT-IR, UV–vis spectra, and ADF-STEM images (PDF)
