# Supplementary material for: Atomic-Scale Imaging of Polymers and Precision Molecular Weight Analysis
Source: J Am Chem Soc. 2024 Dec 4;146(50):34292–7. doi: 10.1021/jacs.4c13812 (PMC11664914; doi:10.1021/jacs.4c13812)
Supplement: Supplementary file 1 — ja4c13812_si_001.pdf [file ja4c13812_si_001.pdf]

Atomic-Scale Imaging of Polymers and Precision Molecular Weight Analysis

Arkadios Marathianos,[a] Alexandros Magiakos,[b] Yisong Han,[c] Ana Sanchez,[c] Richard Whitfield,[d] Jochen Kammerer,[e] Athina Anastasaki,[d] Paul Wilson,[b] Joseph P. Patterson,[f, g] Christopher Barner-Kowollik,[e, h] Evelina Liarou*[b]

[a] Polymer Characterization Research Technology Platform, University of Warwick, Coventry CV4 7AL, United Kingdom

[b] Department of Chemistry, University of Warwick, Library Road, Coventry, CV4 7AL, UK

[c] Department of Physics, University of Warwick, Coventry, CV4 7AL, UK

[d] Laboratory of Polymeric Materials, Department of Materials, ETH Zurich, Zurich 8093, Switzerland

[e] School of Chemistry and Physics, Centre for Materials Science, Queensland University of Technology (QUT), 2 George Street, Brisbane City, QLD, 4000 Australia

[f] Department of Chemistry, University of California, Irvine, Irvine, California 92697-2025, United States

[g] Department of Materials Science and Engineering, University of California, Irvine, Irvine, California 92697-2025, United States

[h] Institute of Nanotechnology, Karlsruhe Institute of Technology (KIT), Kaiserstrasse 12, 76131 Karlsruhe, Germany

Materials

Solvents were purchased from commercial suppliers (Sigma Aldrich / Merck, Fischer Scientific, Appollo Scientific) and used as received. Tris(2-(dimethylamino)ethyl)amine (Me_6Tren) was synthesized according to the literature and stored in an amber vial at 4 °C.[1] Copper(0) in the form of wire (diameter 0.25 mm) was purchased from Scientific Laboratory Supplies. It was purified by immersion in concentrated 37 % HCl for 15 minutes, subsequently rinsed with distilled water and acetone, and dried with compressed air prior to use. 2-(Dodecylthiocarbonothioylthio)-2-methylpropionic acid (DDMAT), ferrocenylmethyl methacrylate, methyl acrylate, 4-Amino-1-butanol (98%), 2,2'-Azobis(2-methylpropionitrile) (AIBN), 1,5,7-Triazabicyclo[4.4.0]dec-5-ene (TBD), N,N'-Dicyclohexylcarbodiimide (DCC) and 4-Dimethylaminopyridine (DMAP) were purchased from Sigma-Aldrich / Merck, 4-Arsanilic acid (≥98%) was purchased from ABCR. In all aqueous solutions, deionized H_2O (DI-H_2O) was used. 2-((butylthio)-carbonothioyl)thio propanoic acid (PABTC) was synthesized according to the literature.[2] Membrane dialysis tubings were obtained from Spectrum Laboratories. The As-acrylamide monomer (4-acrylamidophenyl)arsonic acid) was synthesized according to the literature.[3-5] TEM grids were purchased from Agar Scientific. Dialysis membrane bags were purchased from SpectrumLabs.

Instrumentation & Methods

Size Exclusion Chromatography (SEC)

Aqueous-SEC

All GPC data were recorded on an Agilent Infinity II instrument equipped with differential refractive index (DRI), viscometry (VS) and light-scatter (LS) detectors. The system was equipped with 2 x Agilent PL aquagel-OH Mixed M columns (300 x 7.5 mm) and a 5 mm PL aquagel Guard column. The mobile phase was 0.1 M $NaNO_3$ (aq.)/MeOH 80/20 % v/v and run at a flow rate of 1 mL/min at 35°C. Agilent polyethylene glycol / oxide (PEG/PEO) EasiVials were used to create a third order conventional calibration curve between 427,500 and 194 $g{\cdot}mol^{-1}$. Analyte samples were filtered through PVDF filters with 0.22 μm pore size prior to injection. All sample analysis was carried out using Agilent GPC/SEC Software.

$CHCl_3$-SEC

All GPC data were recorded on an Agilent Infinity II MDS instrument equipped with differential refractive index (DRI), viscometry (VS), dual angle light scatter (LS) and variable wavelength UV detectors. The system was equipped with 2 x PLgel Mixed C columns (300 x 7.5 mm) and a PLgel 5 μm guard column. The eluent was $CHCl_3$ and the samples were run at 1 mL/min at 30 °C. Poly(methyl methacrylate) standards (Agilent EasiVials) were used for the creation of a third order calibration curve between 1,591,000 – 535 $g{\cdot}mol^{-1}$. Analyte samples were filtered through a nylon membrane with 0.22 μm pore size prior to injection. All sample analysis was carried out using Agilent GPC/SEC Software.

THF-SEC

All GPC data were recorded on an Agilent Infinity II MDS instrument equipped with differential refractive index (DRI), viscometry (VS), dual angle light scatter (LS) and variable wavelength UV detectors. The system was equipped with 2 x PLgel Mixed C columns (300 x 7.5 mm) and a PLgel 5 μm guard column. The eluent was THF with 0.01% w/v BHT additive. The samples were run at 1 mL/min at 30 °C. Poly(methyl methacrylate) standards (Agilent EasiVials) were used for the creation of a third order calibration curve between 1,591,000 – 535 $g{\cdot}mol^{-1}$. Analyte samples were filtered through a nylon membrane with 0.22 μm pore size prior to injection. All sample analysis was carried out using Agilent GPC/SEC Software.

^{1}H Nuclear Magnetic Resonance (^{1}H NMR)

^{1}H NMR spectra were recorded on Bruker Avance III HD 300 MHz and HD 400 MHz, and Bruker Avance III 400 MHz (AV400) spectrometers in deuterated solvents obtained from Sigma-Aldrich. Chemical shifts are given in ppm downfield from the internal standard tetramethyl silane. Monomer conversions were determined via ^{1}H NMR spectroscopy by comparing the integrals of monomeric vinyl protons to polymer signals.

Diffusion-Ordered Spectroscopy (DOSY)

All DOSY NMR spectra were acquired using a Magritek Spinsolve 80 Carbon Benchtop NMR spectrometer equipped with a z-axis gradient coil capable of generating a maximum field gradient of 500 mT/m. The number of scans applied were 8-64 and DOSY spectra were interpreted using Magritek Spinsolve version 2.3.6. The MW_{DOSY} was determined according to the literature[6] and the calculations were automatically performed through the online DOSY Molecular Weight Calculator at the University of Warwick.

(https://warwick.ac.uk/fac/sci/chemistry/research/haddleton/haddletongroup/meetthegroup/currentmembers/owentooley/dosymwtcalculator/)

Fourier-Transform Infrared Spectroscopy (FT-IR)

FT-IR spectra were collected on a Cary 630 FTIR Spectrometer (Agilent) with a resolution of ≤ 2 cm^{-1}, scanning in a wavenumber range of 350 and 4000 cm^{-1}. Data was exported using the Microlab software from Agilent.

Atomic Resolution Microscopy:
Annular Dark Field-Scanning Transmission Electron Microscopy (ADF-STEM)

ADF-STEM images were acquired using a probe and image aberration corrected JEOL ARM200F TEM operating at 200 kV, equipped with a Schottky field emission gun (FEG) and a Gatan Orius SC1000 CCD camera. The camera length was 8 cm, the probe current approx. 23 pA and the semi-convergence angle approx. 25 mrad. Throughout the sessions, the emission current was judiciously monitored to verify stability. The samples were prepared at concentrations of 50 and/or 10 μg·mL^{-1} in 0.1 M NaOH, $CHCl_3$ (PFerMMA) or THF (PBAm-Fc), with aliquots of 5 μL being drop casted on graphene oxide (or graphene) supported copper TEM grids. Prior to imaging, the prepared grids were placed in a vacuum station at 40 °C for 24 h, while beam shower was applied for 30 minutes to eliminate contamination/beam damage. For the determination of MW_{STEM}, the intensity of ~100 single As (or Fe for PFerMMA) atoms was initially measured, upon subtraction of their background,

according to the literature.[7] The same background subtraction process was applied to extract the integrated intensity of the individual polymer chains, which was then weighed by the averaged intensity from single As (or Fe) atoms to determine the total number of As (or Fe) atoms per chain. The degree of polymerization (DP_n) for >30 polymer chains was calculated each time. Multiplication of the monomer molecular weight with the DP_n found for each polymer chain yielded the molecular weight of each polymer chain. The average molecular weight of each polymer chain calculated through ADF-STEM atom counting was plotted in a histogram format, with the highest peak denoting the predominant molecular weight distribution (bin centre value used as MW_{STEM}). The images were analyzed using ImageJ and were contrast corrected. The dispersity of polymers was calculated based on standard deviation (σ) according to literature,[8] and is expressed as:

$$Đ = \frac{DP_w}{DP_n} = \frac{\mu^2 + \sum x^2 f(x) - \mu^2}{\mu^2} = 1 + \frac{\sigma^2}{\mu^2}$$

Synthetic Procedures

Synthesis of the arsenic-containing acrylamide monomer 4-(N-acrylamido)phenylarsonic acid (AsAm)

The synthesis of the AsAm monomer was performed according to the literature.[5] Briefly, a round bottom flask equipped with a stirrer bar was charged with 5.1 g KOH (91.8 mmol, platelets) dissolved in 125 mL DI-H_2O. To that, 10.0 g p-arsanilic acid (46.1 mmol) and 14.5 g Na_2CO_3 (137.1 mmol) were added portionwise and the solution was placed in an ice bath (0-1°C). Next, a solution of acryloyl chloride (5.6 mL, 68.9 mmol) in dichloromethane (25 mL) was added in the ice-cold p-arsanilic acid solution and was left to react under stirring for 15 min The aqueous phase was collected and carefully acidified to pH 1 through addition of H_2SO_4 (98%) which resulted in precipitation of product. The precipitate was collected by filtration, washed with cold water and dried in a vacuum oven for 24 h. The As-acrylamide monomer was collected as a white solid. (11.43 g, 49.9 mmol, α=91,8%). **^{1}H NMR** (D_2O/NaOH 0.1M, 400 MHz): δ_H(ppm) = 7.55 (2H, d, J_{HH} = 8.07 Hz, AsCC**H**), 7.11 (2H, d, J_{HH} = 7.95 Hz CC**H**), 6.25 (1H, m, J_{HH} = 10.51, 6.85 Hz, CC**H**), 5.95 (1H, d, J_{HH} = 17.36 Hz, CH**H**), 5.53 (1H, d, J_{HH} = 10.51 Hz, C**H**H); **^{13}C NMR**: 123.12 (**Ar**), 124.29 (H_2**C**=C-), 130.53 (**Ar**) 130.62 (H_2C=**C**-), 133.34 (**Ar**), 134.73 (**Ar**), 168.72 (-**C**=O)

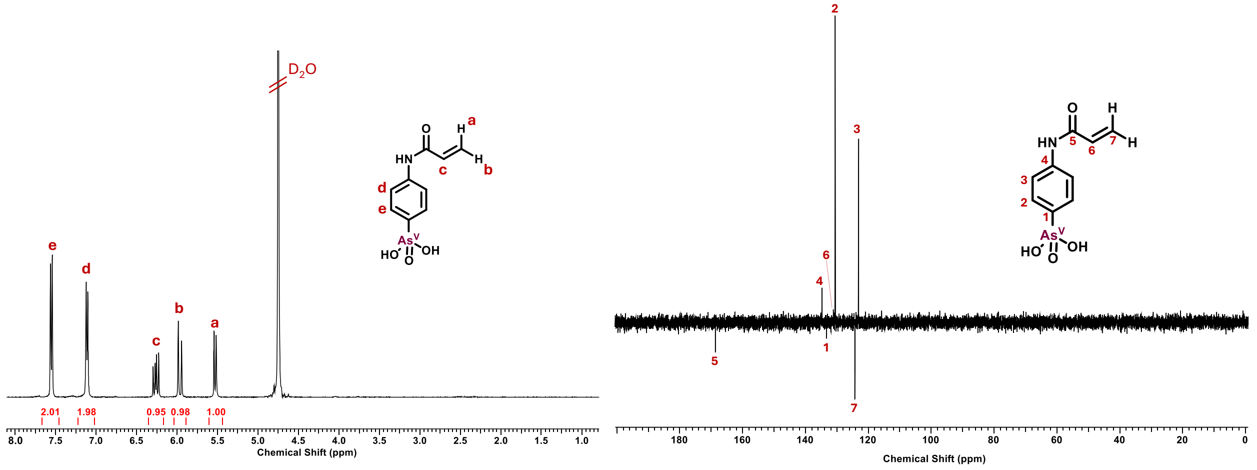

Typical procedure for the synthesis of PAsAm with targeted DP$_n$=50 through Reversible Addition Fragmentation Chain Transfer Polymerization (RAFT) of the arsenic-containing acrylamide (AsAm)

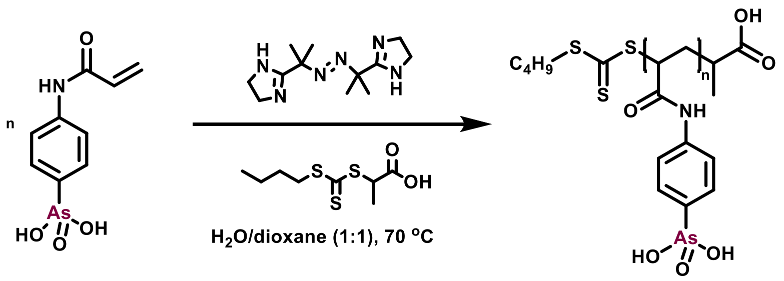

A stock solution containing 20 mg 2,2'-Azobis[2-(2-imidazolin-2-yl)propane]dihydrochloride (VA-044) in 0.5 mL DI-H_2O was prepared (*stock A*), along with a second stock solution containing 100 mg of 2-((butylthio)-carbonothioyl)thio propanoic acid (PABTC) in 1 mL DI-H_2O (*stock B*). An 8 mL vial equipped with a stirrer bar, was charged with 200 mg AsAm, 1 mL DI-H_2O and 1 mL dioxane, and was sonicated for 10 minutes. After sonication, the AsAm solution was placed in an oil bath at 70 °C and was deoxygenated for 15 min, prior to the addition of 12 μL from *stock B* (0.24 mg, 0.05 eq. VA-044) and 35 μL from *stock A* (3.52 mg, 1 eq. PABTC), which was followed by another 15 min deoxygenation through N_2 sparging. The polymerization was left to commence for 15 hours before aliquots for aqueous-SEC and ^{1}H NMR were taken. For the aqueous-SEC characterization, aliquots were prepared in H_2O/0.1M NaOH solutions and filtered through PVDF filters prior to injection. The ^{1}H NMR samples were prepared in D_2O/0.1M NaOH. The polymer was purified through dialysis (MW cut off 1,000-3,000 Da depending on SEC analysis) against DI-H_2O/0.1M NaOH (1:1) for 3 days, with frequent changes of the dialysate, and was collected after freeze-drying. The same conditions were applied for the synthesis of all the PAsAm homopolymers with [M]:[PABTC]:[VA-044]=[DP$_n$]:[1]:[0.05].

Typical procedure for the synthesis of PAsAm through free radical polymerization (FRP) of AsAm.

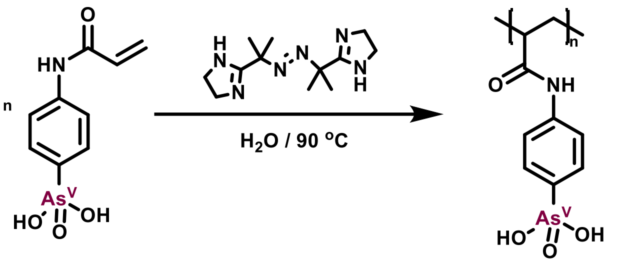

A round bottom flask (50 mL) equipped with a stirrer bar was charged with 500 mg AsAm, 2 mL NaOH 0.1M, 6 mL DI-H_2O and 0.04 mg VA-044 and the reaction solution was deoxygenated through N_2-spraging for 15 min After deoxygenation, the flask was placed in an oil bath at 90 °C and the polymerization was left to commence for 5 h. For the aqueous-SEC characterization, aliquots were prepared in H_2O/0.1M NaOH solutions and filtered through PVDF filters prior to injection. The ^1H NMR samples were prepared in D_2O/0.1M NaOH. The polymer was purified through dialysis (MW cut off 3,000 Da) against DI-H_2O/0.1M NaOH (1:1) for 3 days, with frequent changes of the dialysate, and was collected after freeze-drying. $M_{n,SEC}$= 218,000, $Đ_{,SEC}$=2.3

Typical procedure for the synthesis of PFerMMA$_{10}$ through Reversible Addition Fragmentation Chain Transfer Polymerization (RAFT) of FerMMA

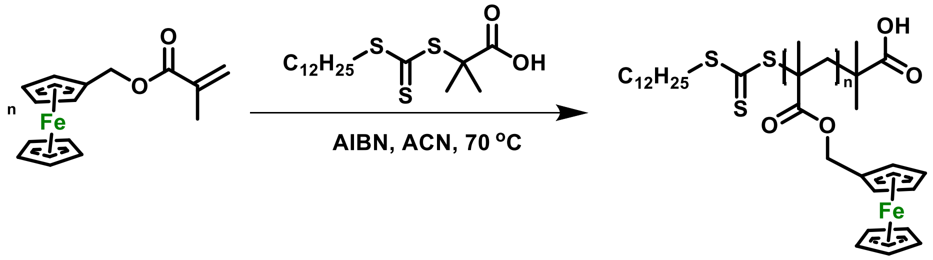

An 8 mL vial equipped with a stirrer bar was charged with 0.29 mg AIBN (0.1 eq., from stock solution in acetonitrile), 100 mg FerMMA (20 eq.) and 6.4 mg DDMAT (1 eq.) in 2 mL acetonitrile. The reaction solution was deoxygenated through N_2 sparging for 15 min and was subsequently placed in an oil-bath at 70 °C for 15 h, before aliquots for SEC and ^1H NMR were taken. The ^1H NMR samples were prepared in $CDCl_3$, while the THF-SEC or $CHCl_3$-SEC samples were prepared in the corresponding solvent. The polymerization reaction was terminated when monomer conversion had reached ~50%. The polymer was purified through (3x) precipitations into hexane, was filtered and was allowed to dry in a vacuum oven at 25°C overnight. DP_n by ^1H NMR=9, $M_{n,SEC}$= 5,900 (PS cal.), 7,100 (PMMA cal.), $Đ_{,SEC}$=1.4

Typical procedure for the synthesis of PMA_{20} through Cu^0 wire-mediated Reversible Deactivation Radical Polymerization (Cu-RDRP) of MA.

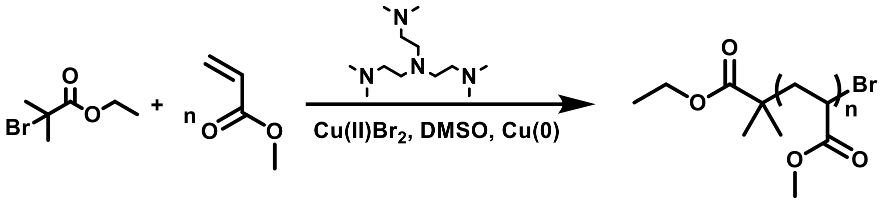

A 20 mL vial was charged with 25 mg $Cu(II)Br_2$ (0.05 eq.), 4 mL DMSO and 106 μL Me_6Tren (0.18 eq.) and was sonicated for ~30 sec until complete dissolution of $Cu(II)Br_2$ and formation of the characteristic green coloured complex. Then, 4 mL (20 eq.) of MA were added, along with 5 cm pre-activated copper wire wrapped around a stirrer bar, and the vial was septum sealed. The reaction solution was N_2-deoxygenated for 15 min before the addition of 323 μL of EBiB (1 eq.), followed by another 5 min deoxygenation. The polymerization was left to commence at ambient temperature for 3h and samples were taken for ^1H NMR (in $CDCl_3$) and THF-SEC characterization, after having been passed through a short column of neutral alumina to remove dissolved copper salts. The polymer was purified through (3x) precipitations in ice-cold $MeOH/H_2O$ (1:5 v/v%) and was allowed to dry in a vacuum oven at 40 °C for 24 h. DP_n by ^1H NMR=20, $M_{n,SEC}$= 1,900, $Đ_{,SEC}$=1.17 (rounded up to $Đ_{,SEC}$=1.2)

Derivatization of PMA_{20}

The derivation of PMA into a Fe-containing polyacrylamide was achieved through 2 post-polymerization reactions with intermediate purification steps, after the synthesis of PMA_{20} via Cu^0 wire-mediated Reversible Deactivation Radical Polymerization (Cu-RDRP) as described above. Specifically:

1. **TBD-catalyzed amidation of PMA_{20} with 4-Amino-1-butanol**

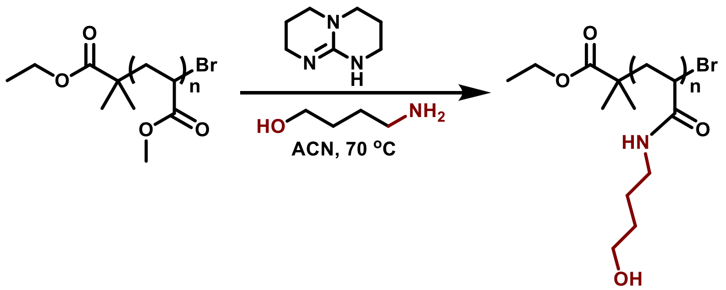

The full amidation of PMA_{20} was adapted from the literature.[9] Specifically, 200 mg of PMA_{20} (1 eq., 0.5 mmol - 20 ester groups on average) were dissolved in 7 mL acetonitrile. In the polymer solution, 146 mg TBD (0.5 eq./ester, 1.05 mmol) and 3 mL 4-amino-1-butanol (~6 eq./ester, 12.6 mmol) were added, the reaction was septum sealed, deoxygenated for 10 min, and subsequently placed in an oil-bath at 80 °C for 48h. Samples were taken for ^1H NMR analysis in DMSO-d_6, FT-IR and SEC in THF. The polymer (polyhydroxybutyl acrylamide, PHBAm) was purified through (2x) precipitations in cold diethyl ether assisted by centrifugation for 15 min and was dried in a vacuum oven at 40 °C for 24 h. Shift towards higher molecular weights ($M_{n,SEC}$=2,400) was verified by THF-SEC, FT-IR verified full shift of the 1730 cm^{-1} peak (C=O, PMA, ester) along with the formation of the 1635 cm^{-1} (C=O, amide) and

1543 cm^{-1} (N-H, bending) peaks attributed to PHBAm, while ^{1}H NMR verified full shift of the PMA methyl protons.

2. Functionalization of PHBAm with ferrocene carboxylic acid through Steglich esterification

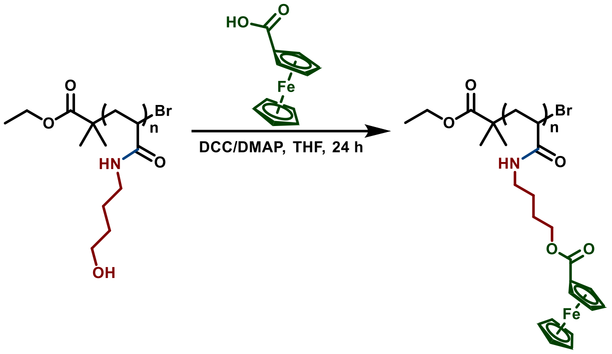

A vial was charged with 5 mL THF, 100 mg PHBAm (1 eq., 0.0416 mmol), 200 mg ferrocene carboxylic acid (1 eq., 0.833 mmol), 189 mg DCC (1.1 eq., 0.917 mmol) and 112 mg DMAP (1.1 eq., 0.917 mmol). The solution was N_2-deoxygenated for 15 min, was subsequently placed in an oil-bath at 40 °C and was left to react for 24h. The solution was filtered to remove the precipitate, and the Fc-functionalized polymer was (x3) washed with H_2O and extracted from dichloromethane. The solvent was removed under reduced pressure and the Fc-functional polymer (PBAm-Fc) was placed in a vacuum oven at 40 °C for 24 h to dry before THF-SEC (RI & UV detection), ^{1}H NMR and FT-IR analysis.

Supporting Figures

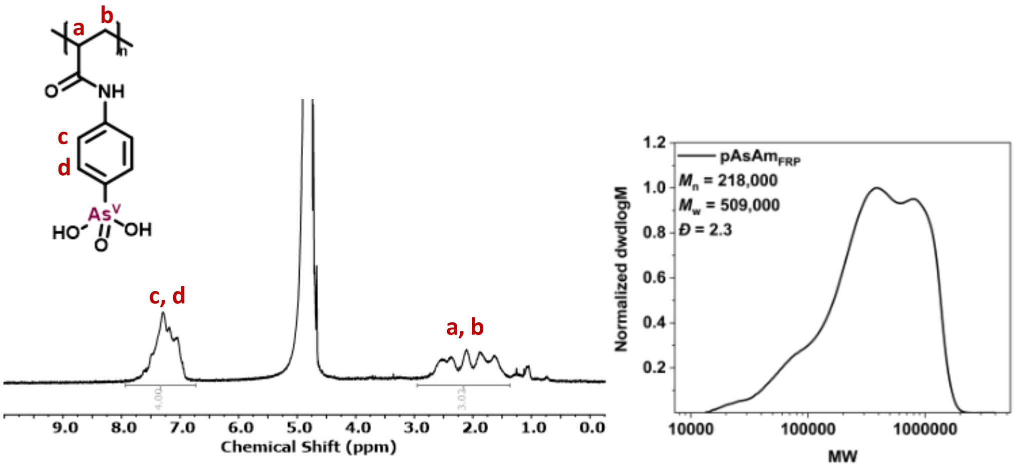

Figure S1. ^{1}H NMR spectrum in D_2O (left) and aqueous-SEC trace (right) for PAsAm$_{FRP}$

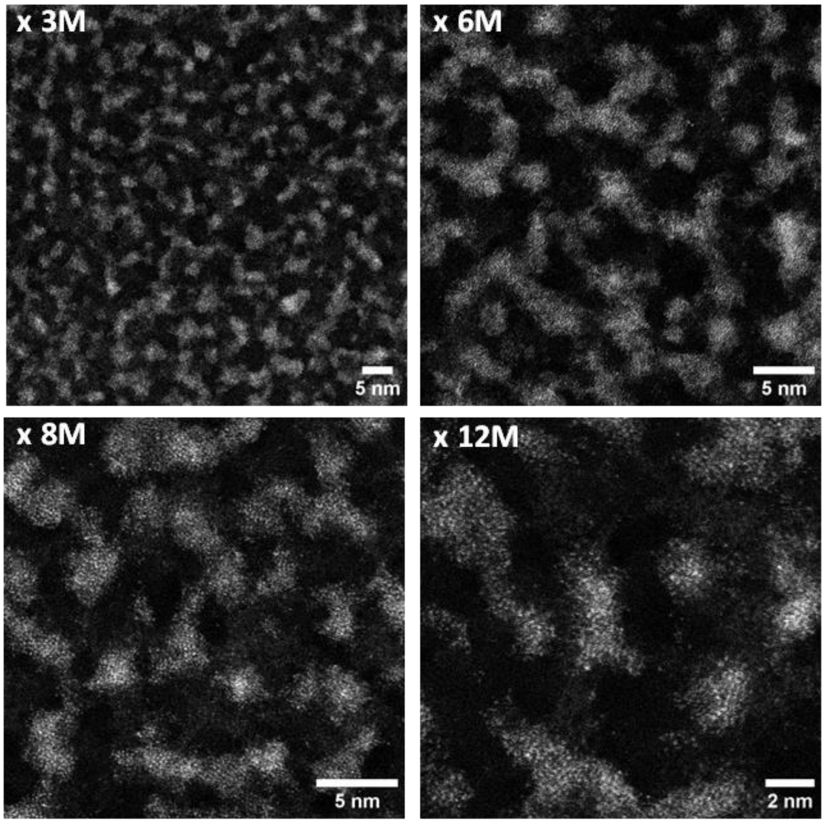

Figure S2. ADF-STEM derived images of $PAsAm_{FRP}$ at different magnification (50 µg·mL^{-1} in 0.1M NaOH), *(post-imaging contrast enhancement)*.

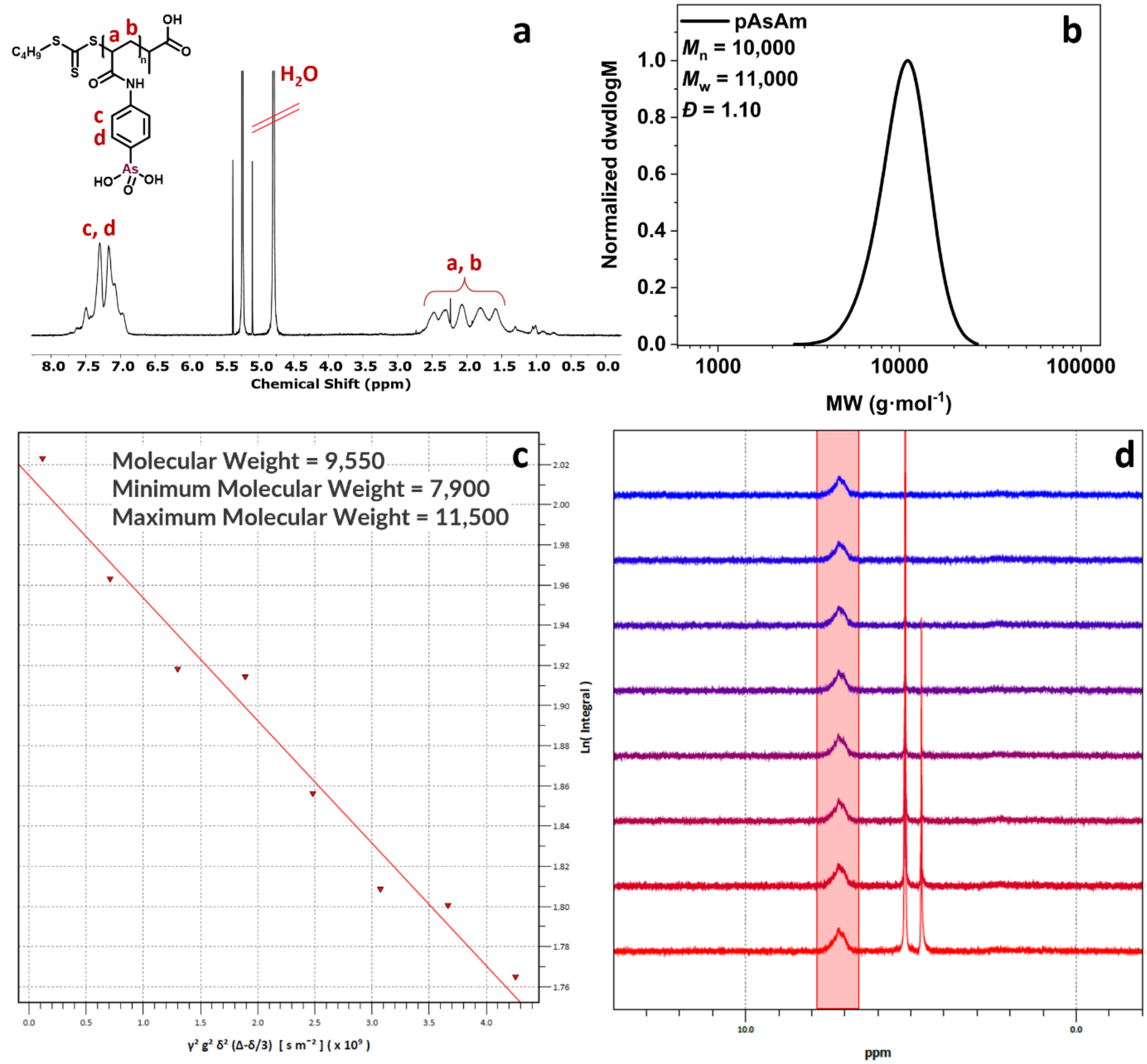

Figure S3. a) ^{1}H NMR in D_2O for the purified $PAsAm_{50}$, b) aqueous-SEC trace for the purified $PAsAm_{50}$, c) Stejskal-Tanner plot showing the ln (integral) as a function of $\gamma^2 g^2 \delta^2 (\Delta - \delta/3)$ as obtained from the Spinsolve software, d) diffusion ^{1}H NMR spectra for the purified $PAsAm_{50}$. Diffusion coefficient, D $=6.103 \cdot 10^{-11}$ m^2s^{-1} as obtained from Spinsolve.

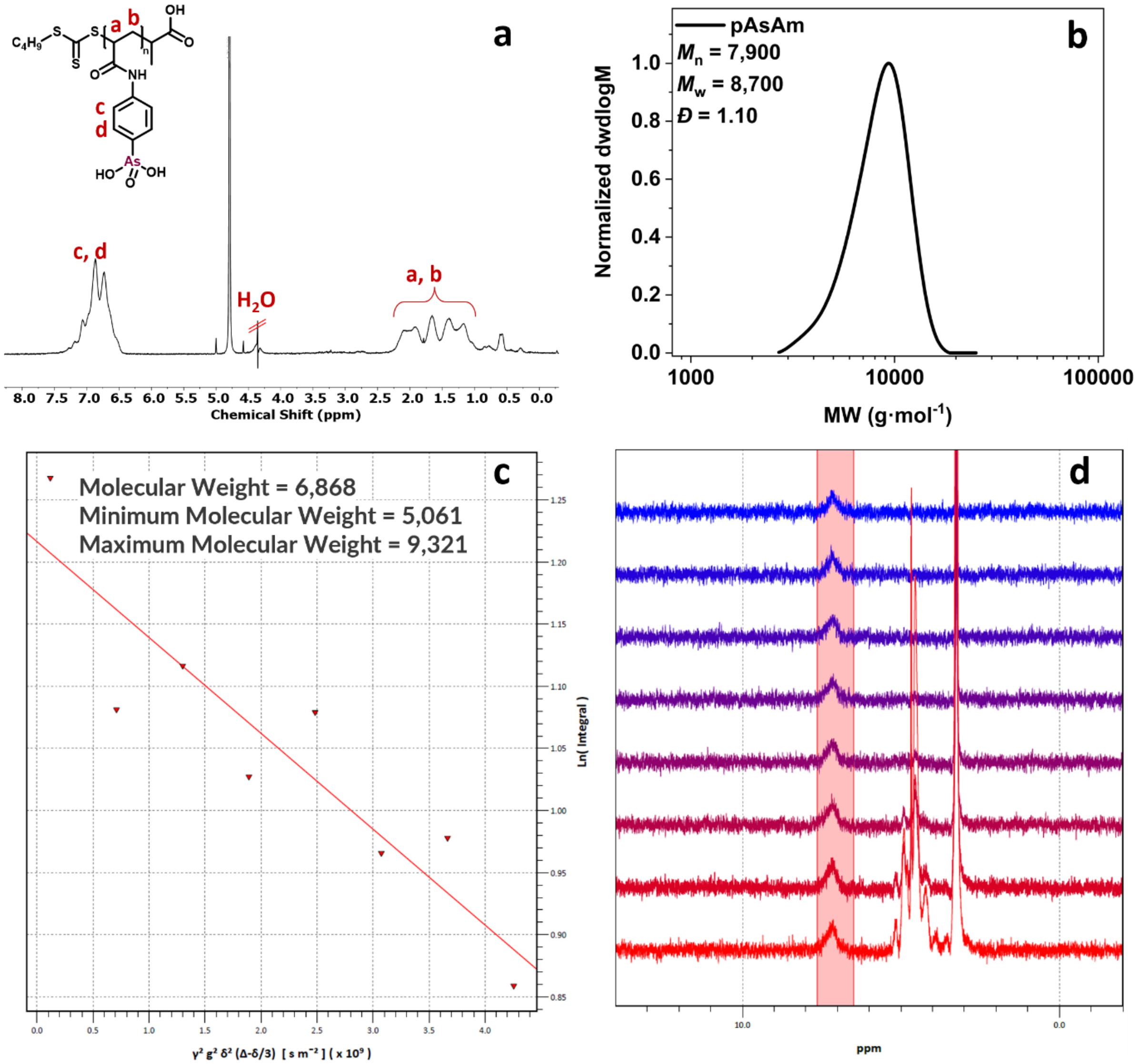

Figure S4. a) ^{1}H NMR in D_2O for the purified $PAsAm_{20}$, b) aqueous-SEC trace for the purified $PAsAm_{20}$, c) Stejskal-Tanner plot showing the ln (integral) as a function of $\gamma^2 g^2 \delta^2 (\Delta - \delta/3)$ as obtained from the Spinsolve software, d) diffusion ^{1}H NMR spectra for the purified $PAsAm_{50}$. Diffusion coefficient, D = $7.714 \cdot 10^{-11}$ m^{2}s^{-1} as obtained from Spinsolve.

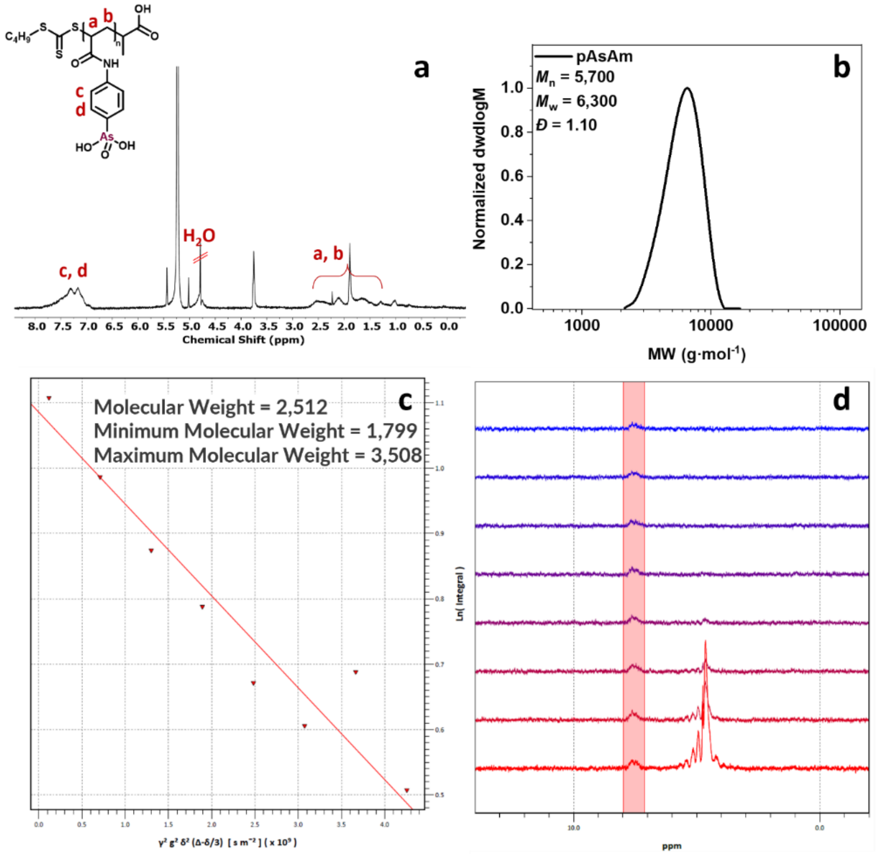

Figure S5. a) ^{1}H NMR in D_2O for the purified $PAsAm_{10}$, b) aqueous-SEC trace for the purified $PAsAm_{10}$, c) Stejskal-Tanner plot showing the ln (integral) as a function of $\gamma^2 g^2 \delta^2 (\Delta - \delta/3)$ as obtained from the Spinsolve software, d) diffusion ^{1}H NMR spectra for the purified $PAsAm_{10}$. Diffusion coefficient, D = $1.406 \cdot 10^{-11}$ m^2s^{-1} as obtained from Spinsolve.

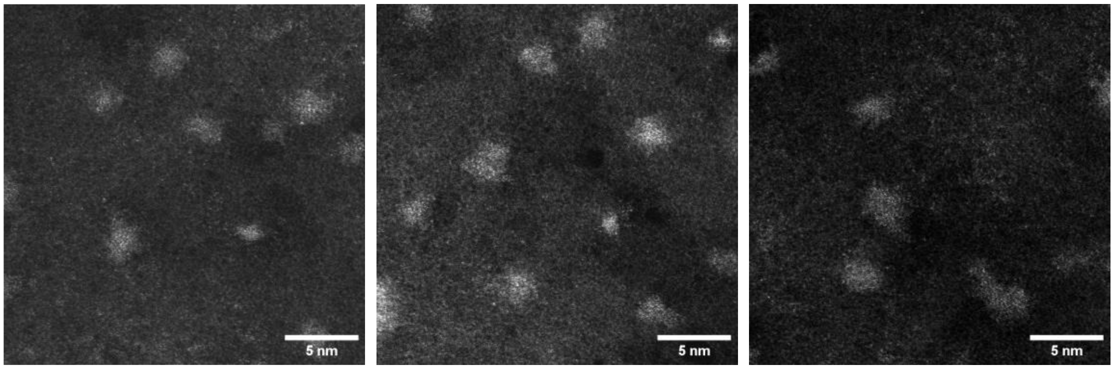

Figure S6. ADF-STEM derived images of $PAsAm_{50}$ at x 8M magnification (50 µg·mL^{-1} in 0.1M NaOH)

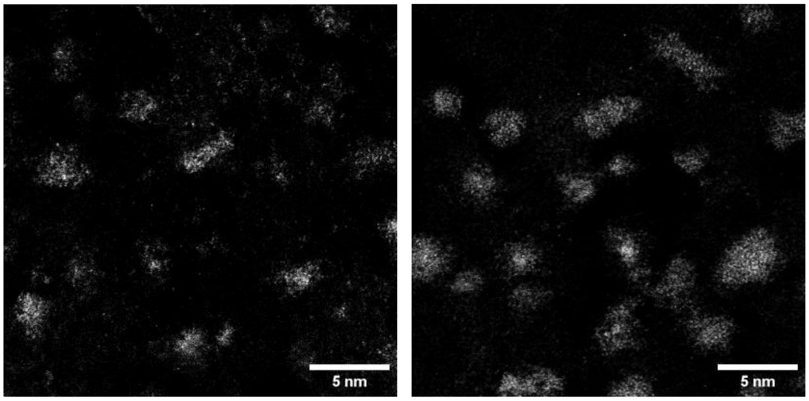

Figure S7. ADF-STEM derived images of $PAsAm_{20}$ at x 8M magnification (50 µg·mL^{-1} in 0.1M NaOH), *post-imaging contrast enhancement*

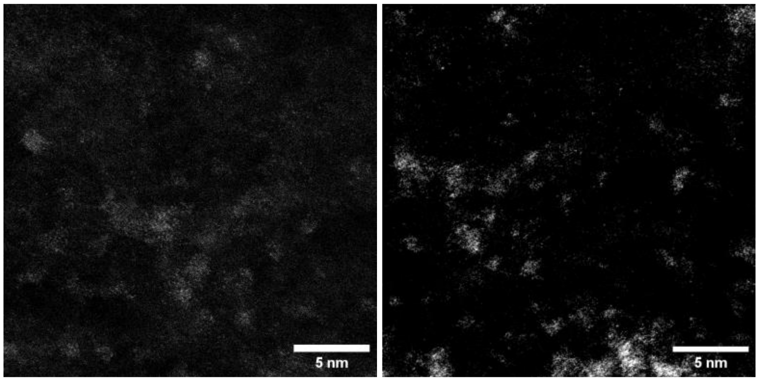

Figure S8. ADF-STEM derived images of $PAsAm_{10}$ at x 8M magnification (50 µg·mL^{-1} in 0.1M NaOH), *post-imaging contrast enhancement*

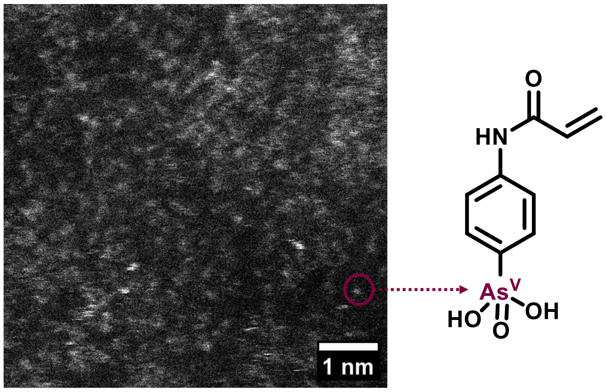

Figure S9. ADF-STEM derived image of the As-containing monomer, AsAm, at x 15M magnification (0.50 mg·mL^{-1} in 0.1M NaOH), *post-imaging contrast enhancement*

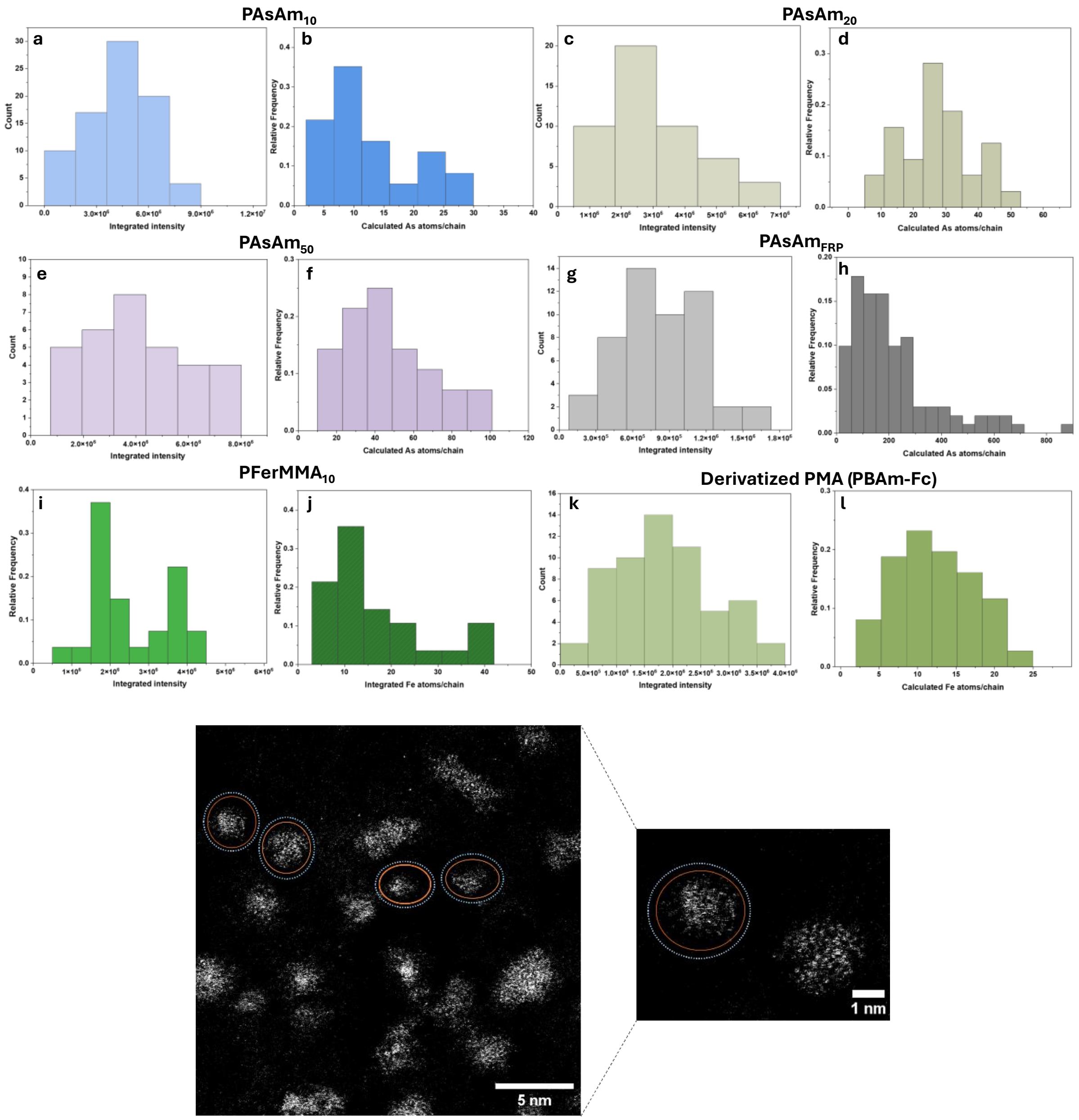

Figure S10. Top: Histograms of integrated intensity corresponding to 1 As atom (a, c, e, g) and 1 Fe atom (i, k), and As (b, d, f, h) and Fe (j, l) atoms per polymer chain (DP_n) calculated based on the corresponding integrated intensities. Bottom: ADF-STEM images (i, ii) demonstrating examples of the regions for the polymer chain intensity calculations with the polymer chain being in the orange circle, and the subtracted background being defined by blue dotted circle (*post-imaging contrast enhancement*).

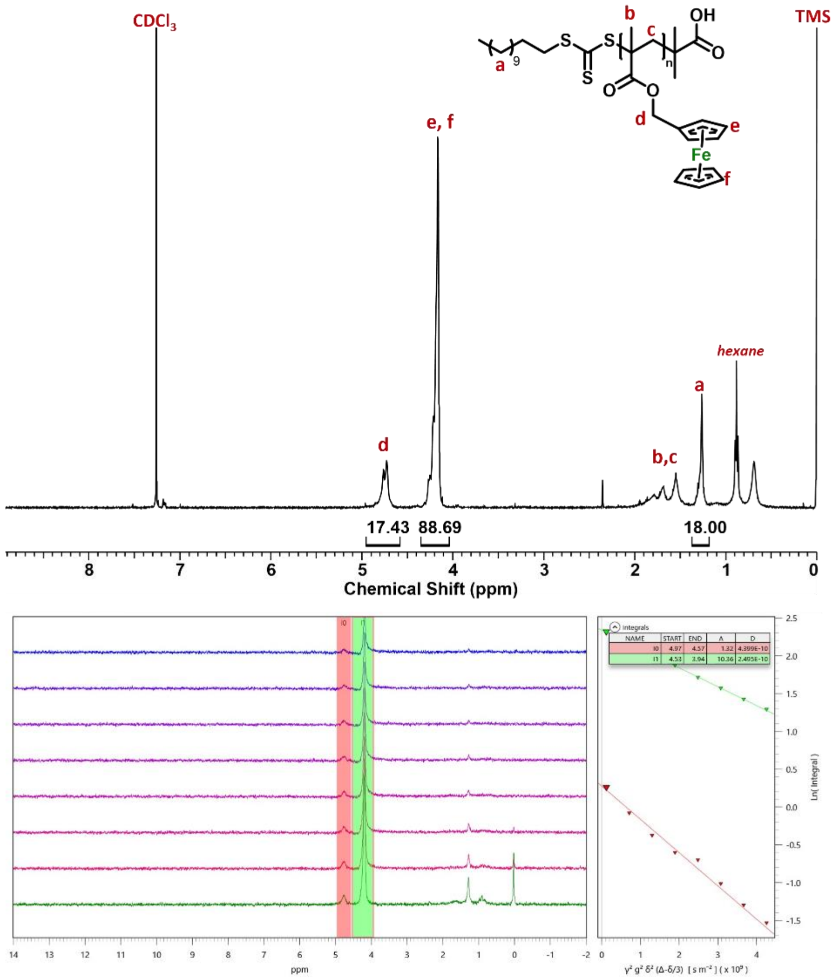

Figure S11. ^{1}H NMR in $CDCl_3$ for the purified $PFerMMA_{10}$ (top) and diffusion ^{1}H NMR spectra for the purified $PFerMMA_{10}$ with the Stejskal-Tanner plot showing the ln (integral) as a function of $\gamma^2 g^2 \delta^2 (\Delta - \delta/3)$ as obtained from the Spinsolve software (bottom). Molecular weight= 3,708 (Minimum Molecular Weight=2,947, Maximum Molecular Weight = 4,667).

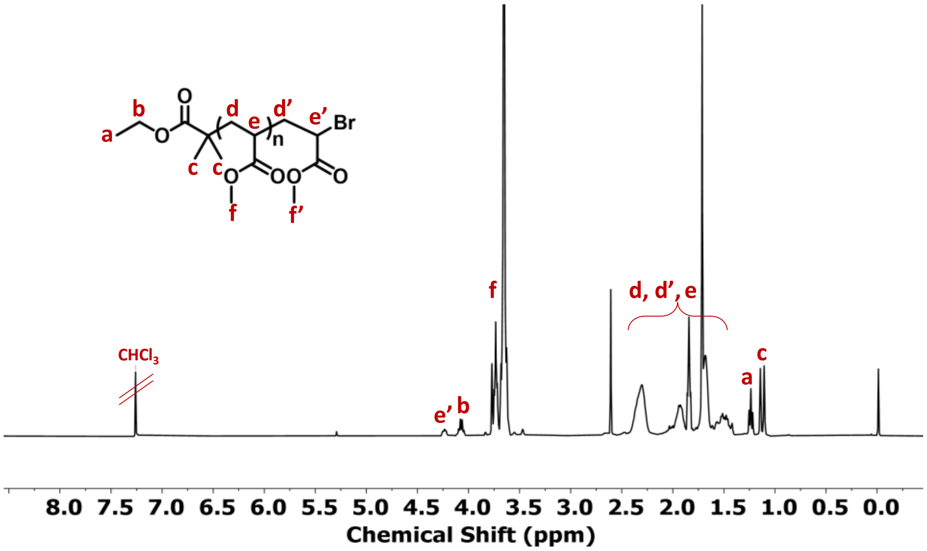

Figure S12. ^{1}H NMR in $CDCl_3$ for the purified PMA_{20} synthesized *via* Cu-RDRP.

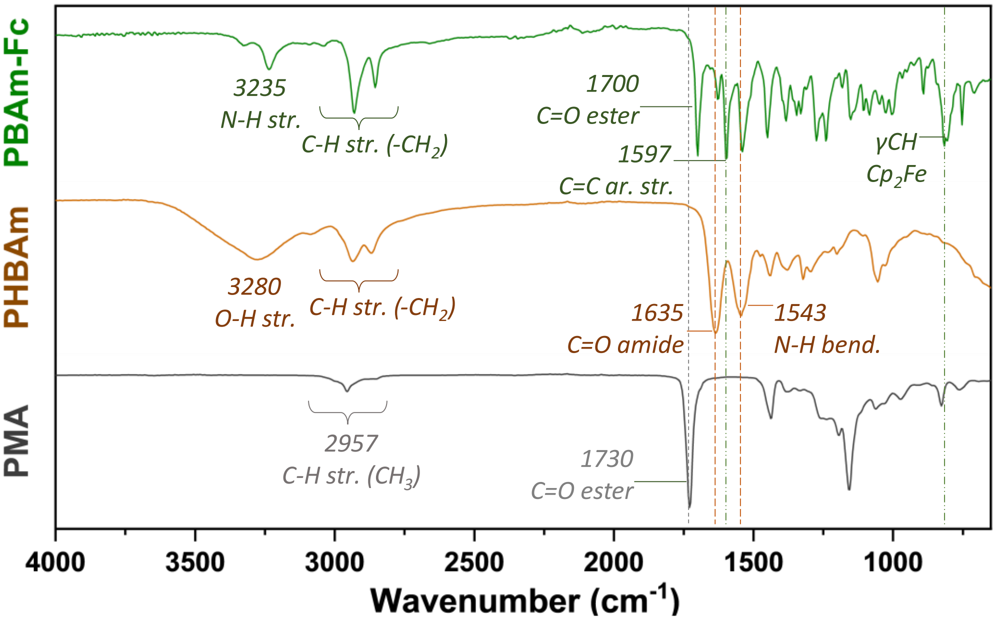

Figure S13. Stacked FT-IR spectra for PMA_{20} (bottom), $PHBAm_{20}$ after amidation of PMA_{20} (middle), and functionalization of $PHBAm_{20}$ to PBAm-Fc (top).

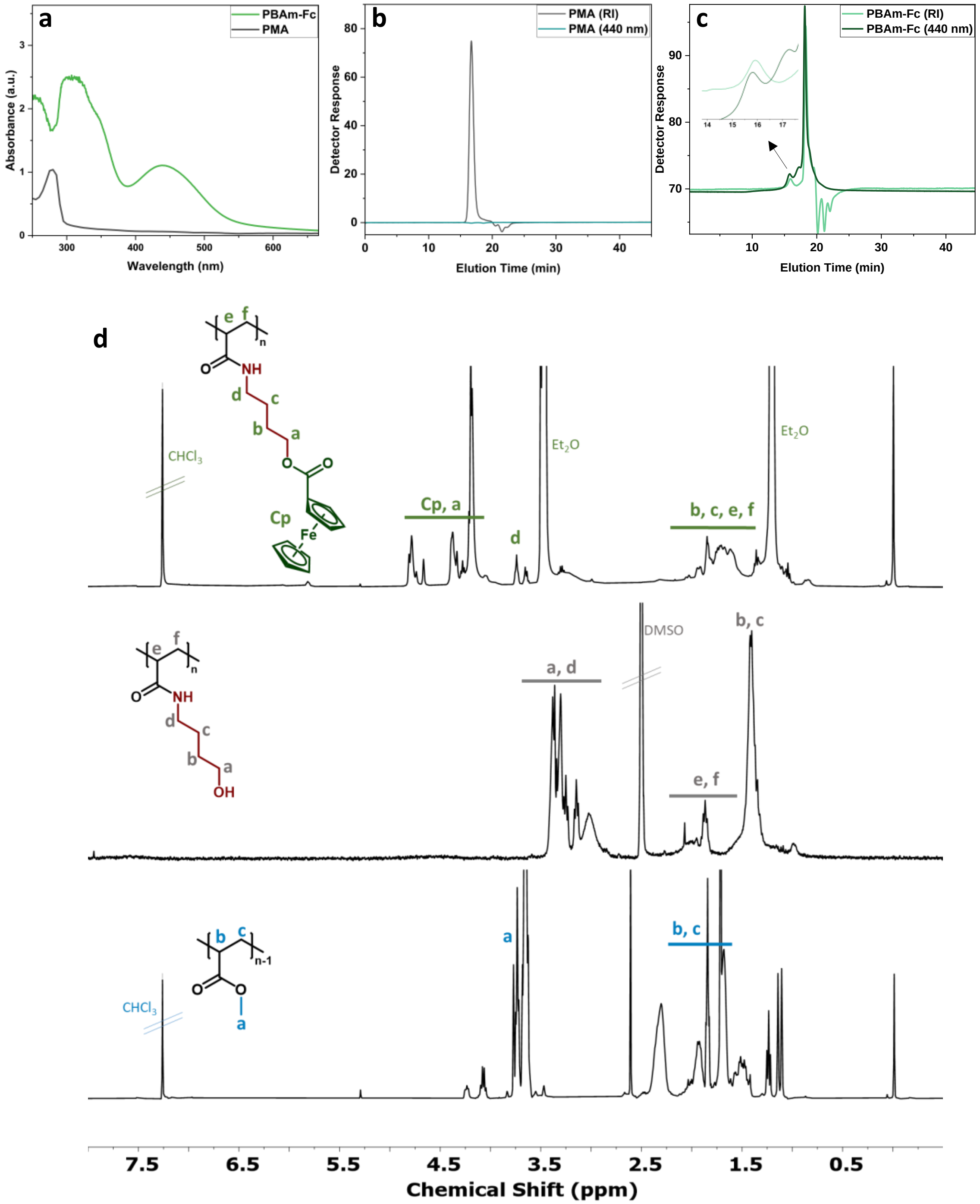

Figure S14. a) UV-Vis spectra of PMA and the Fc-functional PBAm-Fc, SEC-IR and SEC-UV at 440 nm of (b) PMA and (c) the Fc-functional PBAm-Fc showing the detector response after the functionalization of PHBAm to PBAm-Fc and d) stacked ^{1}H NMR spectra for PMA_{20} (bottom), PHBAm (middle) and PBAm-Fc (top).

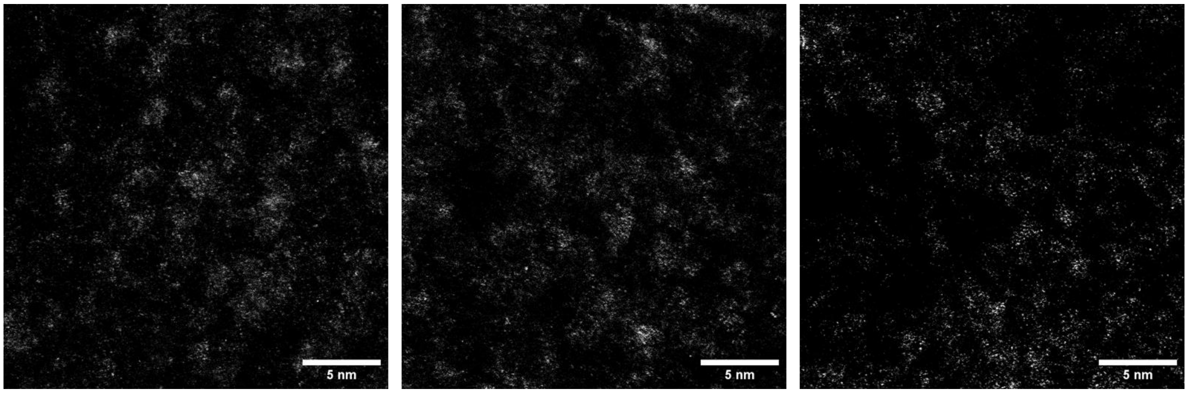

Figure S15. ADF-STEM derived images of PBAm-Fc at x 8M magnification (0.05 mg·mL^{-1} in THF), (*post-imaging contrast enhancement)*

References

(1) Ciampolini, M.; Nardi, N. Five-Coordinated High-Spin Complexes of Bivalent Cobalt, Nickel, and Copper with Tris (2-dimethylaminoethyl) amine. *Inorg. Chem.* **1966**, *5* (1), 41-44.
(2) Ferguson, C. J.; Hughes, R. J.; Nguyen, D.; Pham, B. T. T.; Gilbert, R. G.; Serelis, A. K.; Such, C. H.; Hawkett, B. S. Ab Initio Emulsion Polymerization by RAFT-Controlled Self-Assembly. *Macromolecules* **2005**, *38* (6), 2191-2204.
(3) García-Serrano, J.; Herrera, A. M.; Pérez-Moreno, F.; Valdez, M. A.; Pal, U. Synthesis of novel ionic polymers containing arsonic acid group. *J. Polym. Sci., Part B: Polym. Phys.* **2006**, *44* (11), 1627-1634.
(4) García-Serrano, J.; Pal, U.; Herrera, A. M.; Salas, P.; Angeles-Chavez, C. One-step “green” synthesis and stabilization of Au and Ag nanoparticles using ionic polymers. *Chem. Mater.* **2008**, *20* (16), 5146-5153.
(5) Tanaka, J.; Tani, S.; Peltier, R.; Pilkington, E. H.; Kerr, A.; Davis, T. P.; Wilson, P. Synthesis, aggregation and responsivity of block copolymers containing organic arsenicals. *Polym. Chem.* **2018**, *9* (13), 1551-1556.
(6) Tooley, O.; Pointer, W.; Radmall, R.; Hall, M.; Beyer, V.; Stakem, K.; Swift, T.; Town, J.; Junkers, T.; Wilson, P.; et al. MaDDOSY (Mass Determination Diffusion Ordered Spectroscopy) using an 80 MHz Bench Top NMR for the Rapid Determination of Polymer and Macromolecular Molecular Weight. *Macromol. Rapid Commun.* **2024**, *45* (8), 2300692.
(7) Han, Y.; He, D. S.; Liu, Y.; Xie, S.; Tsukuda, T.; Li, Z. Y. Size and Shape of Nanoclusters: Single-Shot Imaging Approach. *Small* **2012**, *8* (15), 2361-2364.
(8) Harrisson, S. The downside of dispersity: why the standard deviation is a better measure of dispersion in precision polymerization. *Polym. Chem.* **2018**, *9* (12), 1366-1370, 10.1039/C8PY00138C.
(9) Van Guyse, J. F. R.; Verjans, J.; Vandewalle, S.; De Bruycker, K.; Du Prez, F. E.; Hoogenboom, R. Full and Partial Amidation of Poly(methyl acrylate) as Basis for Functional Polyacrylamide (Co)Polymers. *Macromolecules* **2019**, *52* (14), 5102-5109.
